# Postural Adjustments and Kinematic Index Finger Features in Frail Older Adults under Different Equilibrium Constraints

**DOI:** 10.3390/healthcare9080921

**Published:** 2021-07-21

**Authors:** Bianca Callegari, Alexandre Kubicki, Ghislain Saunier, Manuela Brito Duarte, Gizele Cristina da Silva Almeida, Bruno Mazziotti Oliveira Alves, César Ferreira Amorim, Daniela Rosa Garcez, Givago da Silva Souza, France Mourey

**Affiliations:** 1 Laboratory of Human Study Motricity, Federal University of Pará, Av. Generalíssimo Deodoro 01, Belém 66073-000, PA, Brazil; manubritd@gmail.com (M.B.D.); g.c.fisioterapia@gmail.com (G.C.d.S.A.); 2 INSERM U1093, Cognition Action et Plasticité Sensori-Motrice, Université de Bourgogne Franche-Comté, Dijon, France and FiKiP Hôpital Nord Franche-Comté, 2 rue du Docteur Flamand, 25200 Montbéliard, France; alexandre.kubicki@hnfc.fr; 3 Laboratorio de Cognição Motora, Departamento de Anatomia, Universidade Federal do Pará, Rua Augusto Corrêa 01, Belém 66075-110, PA, Brazil; ghislain@ufpa.br; 4 Doctoral and Master’s Program in Physical Therapy, UNICID, 448/475 CesárioGaleno St., São Paulo 03071-000, SP, Brazil; brunomazziotti@hotmail.com (B.M.O.A.); cezar@emgsystem.com.br (C.F.A.); 5 Département des Sciences de la Santé, Programme de physiothérapie de l’université McGill offert en extension à l’UQAC, Chicoutimi, QC G7H 2B1, Canada; 6 Physical Therapy and Neuroscience Departments, Wertheims’ Colleges of Nursing and Health Sciences and Medicine, Florida International University (FIU), Miami, FL 33199, USA; 7 Hospital Universitário Bettina Ferro de Souza, Universidade Federal do Pará, Rua Augusto Corrêa 01, Belém 66075-110, PA, Brazil; drgarcez@gmail.com; 8 Institute of Biological Sciences, Federal University of Pará, Rua Augusto Corrêa 01, Belém 66075-110, PA, Brazil; givagosouza@ufpa.br; 9 INSERM U1093, Cognition Action et Plasticité Sensori-motrice, Université de Bourgogne Franche-Comté, 21078 Dijon, France; france.mourey@u-bourgogne.fr

**Keywords:** postural adjustments, frailty, stability, surface electromyography

## Abstract

**Background:** Anticipatory postural adjustments (APAs) are significantly affected by age and may represent restrictions on functional independence. Previous studies in young adults have already highlighted that changing postural stability (i.e., seated vs. upright posture) affects the motor planning and APAs. In frail older adults (FOAs), the effect of these different conditions of postural stability have not yet been established, and the present study aimed to disentangle this issue. **Methods:** Participants executed an arm-pointing task to reach a diode immediately after it turned on, under different conditions of stability (seated with and without foot support and in an upright posture). A kinematic profile of the index finger and postural electromyographic data were registered in their dominant-side leg muscles: tibialis anterior, soleus, rectus femoris, and semitendinosus. **Results:** The main finding of this study was that the adopted posture and body stabilization in FOAs did not reflect differences in APAs or kinematic features. In addition, they did not present an optimal APA, since postural muscles are recruited simultaneously with the deltoid. **Conclusion:** Thus, FOAs seem to use a single non-optimal motor plan to assist with task performance and counterbalance perturbation forces in which they present similar APAs and do not modify their kinematics features under different equilibrium constraints.

## 1. Background

During arm movements, self-induced body perturbations are expected by the central nervous system (CNS) and anticipatory strategies are generated [1,2] both to counteract these perturbations and maintain dynamic balance but also to create necessary momentums to initiate movements toward the target [3,4,5].These strategies are known as anticipatory postural adjustments (APAs) and are programmed as a feedforward control mechanism, consisting of changes in the activity of postural muscles, 100 to 150 ms prior to the focal muscle [1].

APAs are importantly affected by age [6,7,8] and may represent an important restriction for functional independence in older adults. Similar APA amplitude between young and healthy older adults was previously described, but aged adults have delayed APAs, even later than the onset of prime mover muscles [6,9,10,11]. In frail older adults (FOAs), APAs were delayed and reduced [12,13]. Investigations into APAs in this population are rare and mostly focus on center of pressure (COP) displacement [12,14].

Although it was not investigated, neither in normal nor in pathological elderly, the posture adopted while performing various types of pointing movements also affect APA behavior [15,16]. Recently, we demonstrated that when postural stability is manipulated, young adults modify their motor planning, changing both the focal movement and the APA features. We modified the degrees of postural stability, using two seated postures (i.e., with and without foot support) and a standing posture, while subjects performed an arm pointing task. We found an increase in the reaction time and movement duration when the body was less stabilized (standing posture), which reflects a more challenging task and complex motor plan. APAs were present even when the body was stable (seated with foot support), which suggests an additional APA role, independent of postural stability, beyond the feedforward control of the other body parts (i.e., to accelerate or to facilitate the pointing movement) [4]. Therefore, young adults adopt APAs to improve the task performance and kinematic features even with a stabilized body.

Accordingly, the present study was the first to investigate if postural stability manipulation (i.e., standing and sitting) affects APAs in FOA subjects in the same manner as it was previously demonstrated for healthy subjects. FOAs are people with increased risk of fall [12,13], which is linked, among other things, with the balance function including the management of self-paced perturbations [17]. It is then interesting to investigate their APA programming, manipulating body stability, to understand how this population controls their balance in advance of a predictable perturbation. To address this issue, we utilized an arm-pointing task paradigm, where we instructed the participants to execute the task to reach a diode immediately after it turned on, under different conditions of stability. We evaluated the sequence of muscle activation adopted by FOAs, among the different positions and body stability context.

We hypothesized that if anticipatory electromyographic activation is observed, then FOAs adopted APAs. We further hypothesized that if the main concern of these APAs is to compensate the postural perturbation, then APAs will be attenuated or present altered patterns under increased body stability (i.e., seated with feet support). On the other hand, if an APA serves the preparation of a forthcoming upper limb movement (i.e., facilitate the movement to perform the task), similar APA patterns, independent of the equilibrium constraint, would be present.

## 2. Methods

### 2.1. Study Design

This study was a cross-sectional study, conducted in FOAs and followed the guidelines of the Strengthening the Reporting of Observational Studies in Epidemiology (STROBE statement). The study was approved by the Ethics Committee of Federal University of Para (report #1384907). The subjects were informed about the procedures, and written informed consent forms agreeing to their participation were obtained. All experiments were performed in accordance with the tenets of the Helsinki Declaration.

### 2.2. Subjects

A total of 10 FOAs, who participated in the present study after giving their written consent, are described in Table 1. Frailty can be characterized as a decline in the physiological capacity of multiple organ systems, leading to increased vulnerability to stressor events [18]. The participants were all males with no neurological or muscle disorders and were right-handed. FOAs were submitted to a geriatrician diagnosis according to the clinical features of this syndrome [18]. In addition to the medical diagnoses, they were eligible to participate as subjects if three or more of the following criteria were present: unintentional weight loss, self-reported exhaustion, weakness, slow walking speed, and low physical activity. The exclusion criteria were suffering from a neurological syndrome or peripheral neuropathy, having recent orthopedic or traumatic injuries (<1 year), and/or cognitive impairments with loss of intellectual capacity that compromised the understanding and execution of the task.

### 2.3. Experimental Setup and Protocol

**Figure 1:**
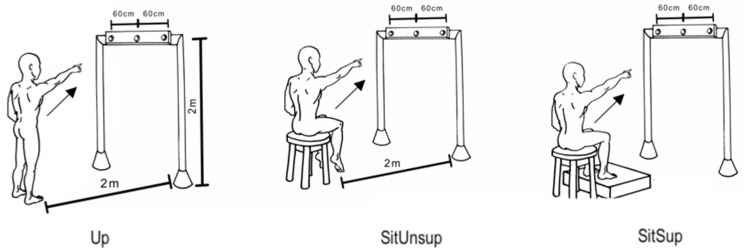
Experimental pointing task. A view of experimental setup for the task displaying a subject in the final posture and the diode of the bar, placed precisely in front of each subject’s right shoulder. Participants were asked to point their index finger at the diode as soon as it turned on.

Three postures were employed to manipulate the postural stability: (i) barefooted upright position; (ii) seated with feet support (Sit Sup); (iii) seated without foot support (SitUnsup). For seated postures, subjects had no back support, and kept 30% of their thigh length in contact to the seat (i.e., from the head of their femurs to the intra-articular line of their knees). We adjusted the height of the seat to make sure their feet would not touch the ground in the SitUnsup posture (Figure 1).

Subjects were instructed to point to a central diode attached to a horizontal bar fixed in their front, with their index finger as soon as it turned on. Protocol followed previous recommendations [4]. Subjects performed ten randomized block trials in each posture, with a 5 min interval between them.

### 2.4. Kinematic and Electromyographic Recording

Four reflective markers were attached to their right upper main joints (i.e., index, wrist, elbow, and shoulder) and kinematic data was recorded using a three-dimensional motion analysis system (Simi Motion) using three cameras at a 120 Hz sampling frequency.

The study assessed surface electromyographic (EMG) data, from their dominant-side leg muscles using disposable self-adhesive electrodes (Medtrace^®^ 200—Kendall, Covidien, Canada): tibialis anterior (TA), soleus (SOL), rectus femoris (RF), semitendinosus (ST), and deltoids anterior (DEL). Lower limb muscles were chosen due to the fact of their role in the ankle and knee control, especially during sitting without foot support, and previously being demonstrated to be related to APAs [4]. Such data were obtained by using two EMG equipment (EMG System ^®^, EMG System do Brasil, São José dos Campos, Brazil) using a sampling rate of 2 KHz per channel and a pass-band ranging from 20 to 500 Hz. EMG signals were amplified (4.000) and digitized with a 16 bit resolution. Instrumentation and sensor location followed the international guidelines of the Surface Electromyography for Non-Invasive Assessment of Muscles [19].

### 2.5. Data Analysis

All kinematic and EMG analyses were previously described by the authors [4] and are summarized below.

### 2.6. Kinematic Characteristics

Kinematic data referring to axis *x*, *y*, and *z* were filtered by a 10 Hz low-pass, second -order bidirectional Butterworth filter. Tzero was defined on the finger trajectory and corresponded as the moment when the tangential velocity of this marker reached 5% of the maximum velocity [5]. The total movement duration (MD) was considered the time interval between the Tzero moment and the trial end, when the index finger stopped pointing to the LED. Movement velocity (MV) and reaction time (RT) were also evaluated. The differences in the index tangential velocity profile in function of the participant postures were also calculated. To do so, we considered the fraction of movement time required to reach peak velocity, which is known as the ratio of acceleration time to total movement duration (ACC/MD).

### 2.7. EMG Data

By using the MATLAB program, we synchronized and analyzed data offline. All EMG signals were rectified (RMS) and filtered by a 100 Hz low-pass, second-order Butterworth filter. Individual trials were displayed offline on a monitor screen.

All data were aligned related to Tzero and EMG signals were integrated from −150 with respect to Tzero (∫EMG 150) in order to quantify anticipatory changes in muscle activity prior to movement. What was later corrected for background activity was defined as the integral from −500 to −450 ms with respect to Tzero (∫EMG 50) as the following:

∫EMG = ∫EMG 150 − 3*∫EMG 50

To allow for comparisons, integrated EMG (EMGi) data were normalized by the peak muscle activity across all postures within an experiment for each muscle and for each subject [15,20]. As a result of the normalization, all EMGi data are within the range from +100 to −100, with the positive values indicating an activation of the muscle and negative values indicating inhibition. Finally, we calculated the average of the data obtained in each subject’s trials within a series of postures.

**Figure 2:**
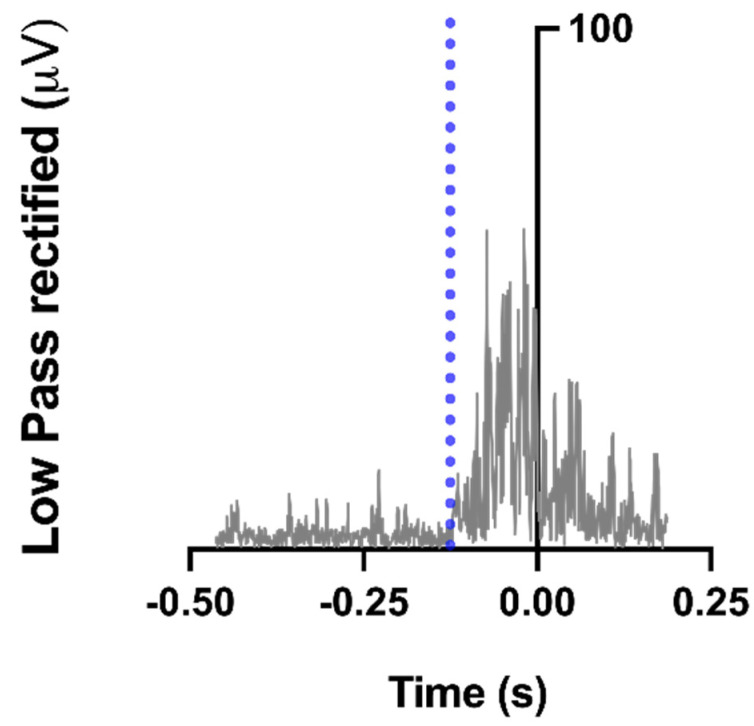
Example of a muscle onset detection. Semitendinosus rectified, low-pass filtered activity of a typical participant, recorded during a single trial. The vertical blue, dashed line indicates muscle onset (EMG amplitude was greater than the mean plus 2 SD of its baseline value, measured from −500 to −450 ms).

We detected muscle latency in a time window ranging from −450 ms to +200 ms in relation to Tzero by using a combination of a computer algorithm and visual inspection of the averaged trials. Timing of the activation or deactivation for a specific muscle was considered the moment after which, for at least 50 ms, its EMG amplitude was greater (activation) or smaller (deactivation) than the mean of its baseline value, measured from −500 to −450 ms, plus or minus 2 SD [15,20]. Figure 2 depicts the example of a muscle onset detection.

After having the onset of each trial, we calculated the timing of each muscle activation with reference to DEL onset [12]. This EMG synergy allowed us to clearly identify the muscles mainly involved in APA within each group, without misinterpreting factors associated with electro-mechanical delays.

### 2.8. Statistical Analysis

The sample size was calculated using 80% statistical power and 95% confidence interval. The mean and standard deviation for the peak velocity (ms) for executing the task was estimated as 3.47 ± 00.5. A minimum required sample size of 6 individuals was calculated. Statistical procedures were carried out in RStudio (R version 3.3.2, R Core Team (2016). The Shapiro–Wilk test was performed to test the data normality. A repeated-measures ANOVA was performed with body posture (sitting with support, sitting without support, and standing) as a factor. Post hoc analyses were done with Tukey’s HSD tests when necessary. For all these statistical treatments, the significance level was set at *p* < 0.05.

## 3. Results

### 3.1. Kinematic Characteristics

Table 2 summarizes the kinematic characteristics. Finger displacement showed a similar RT, MD, MV, and ACC/MD among the three postures in FOAs, revealing that temporal parameters of upper-limb movements were not affected by postural conditions, and their spatial features remained similar.

### 3.2. Muscle Activation Timing between Postures

**Figure 3:**
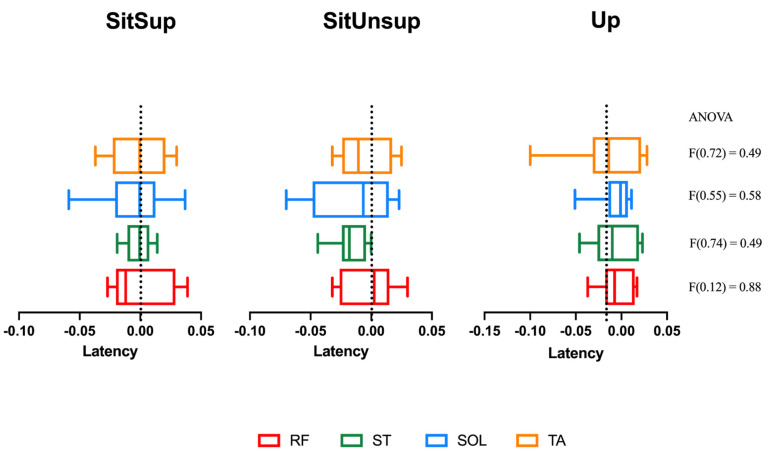
Lower limb muscles’ latency related to deltoids onset. Up: upright posture; SitSup: sit posture + foot contact support; SitUnsup: sit unsupported posture. Muscles: ST: semitendinosus; RF: rectus femoris; SOL: soleus; TA: tibialis anterior. Data expressed with a black line = median; box = 25 and 75 percentiles; whiskers = minimum and maximum values.

The usual activation pattern described in the literature [21], in which muscles begin the anticipatory activation from distal to proximal muscles, was not observed in FOAs. As shown in Figure 3, postural muscles presented simultaneously activation between all the recorded posture, and no posture effect was observed (Figure 3). Supplementary Materials are available.

### 3.3. Muscle Activation Magnitude in the APA Period between Postures

Table 3 summarizes normalized integrated electromyographic activity (EMGi). SOL, TA, and RF showed similar behavior and presented no relevant posture effects among the three postures. ST showed a main posture effect (F(2, 2.54) = 0.016), and the post hoc test showed the highest APA integral in the SitUnsup posture, compared to the others (*p* < 0.05).

## 4. Discussion

In this paper, we aimed to investigate the APA features in FOA subjects when the condition of stability was modified by the adopted posture to realize a pointing task. Our main finding showed that the manipulation of the equilibrium context did not reflect modifications for the subjects, neither in the kinematic features to execute the task nor in the motor plans (i.e., APAs). Besides the subjects displaying the same pattern of muscle activation among the different positions, no differences showed up in APAs under different equilibrium contexts. According to our hypothesis, if APAs were not altered under increased body stability, they were then functionally link to the task, to ensure an active role in task goal achievement (i.e., rather than acting only to stabilize the COM).

### 4.1. Kinematic Features of the Upper Limb

The kinematic data revealed that the FOAs faced similar difficulties in performing the pointing task in the different postures, since no increases in RT, MD, and MV or modification in the velocity profile was observed. These parameters are classically described as indicators of higher index of task difficulty when RT and MD increase and ACC/MD decrease [22]. This behavior is interesting, since it was previously demonstrated that in healthy young subjects, kinematic features are commonly modulated according to their stability constraint. In the literature, healthy adults presented higher RT and MD and longer deceleration duration of the pointing movement when adopting the less stable posture [4]. It seems that for young adults, equilibrium constraints were considered to plan the movement and provoked greater difficulty to plan the task (i.e., smaller peak of velocity), acting as a subtask (balance). In our study, FOAs seemed to have greater difficulty, no matter the stability manipulation, and no differences regarding the stability were considered to plan the task.

### 4.2. Muscles Activation Features

Our results support the hypothesis that FOAs conserved the same motor programming among the different positions, as the CNS was more focused on the accuracy and execution of the task than in the postural preparation. Therefore, they presented a global activation, with almost no selectivity between proximal and distal muscles. Contrary to what was previously reported in healthy young subjects, no difference in APAs were observed independently of the posture adopted, and no specific order of activation between the proximal and distal lower limb muscles could be described. In young adults, previous results demonstrated that an unstable posture (i.e., upright position) was associated with APAs in a different pattern compared to a sitting posture. Distal leg muscles (SOL and TA) followed by the proximal thigh muscles (ST and RF) in the Up posture were described in this population, while a reverse order was revealed during seated posture [4]. Considering this, we may say that FOAs have lost this programing ability, and this may explain the less efficient kinematic profile adopted by this population, no matter the posture stability.

We observed that distal muscles presented activation of TA and deactivation of SOL during APA intervals, as previous described by Fautrelle et al. [23]. This means that SOL has reduced its activation during the APA phase, and we observed negative values in activation amplitude. Proximal muscles, RF and ST, presented the same activation behavior than TA and higher amplitudes in EMG during APA periods, compared to its baseline. However, the magnitude of this activation/deactivation was only directly affected by the postural stability in ST. Higher APA magnitudes were previously demonstrated in healthy subjects under more unstable constraints [4]. In FOAs, however, only the ST suffered a postural effect whit higher EMGi in a SitUnsup posture when compared to both Up and SitSup postures. The ST is one of the hamstrings muscles that cross and act upon hip and knee joints, so they are biarticular muscles. In standing posture, literature shows that the proximal muscle ST is related to its role in the hip extension to counteract the forces on the pelvis produced by the reaching task and producing a trunk forward tilting movement. On the contrary, the seated subjects have the pelvis stabilized by the natural postural chain, and the ST acts as the knee flexor instead of hip extensor, especially with unsupported feet. Indeed, forward ankle displacement in this posture was previously demonstrated in healthy young [4]. This may explain the difference in ST amplitude in our results.

In summary, contrary to what was previously described in healthy young subjects, who presented different APA patterns under different stability levels, in FOAs, the adopted posture and body stabilization did not reflect differences in APAs or kinematic features, seeming that they present a less efficient programing ability related to postural control.

Our study has a potential limitation. When we calculated the activation rate for each muscle in each posture (which corresponded to the percentage of trials showing significant muscle activation), FOAs presented an average of only 45% of trials with burst (i.e., onset in the APA period). This average means that APAs were not present in the majority of trials, and one can speculate about the consistency of the data. On the other hand, we believe that this strongly reinforces the lack and inefficiency in FOAs to adopt anticipatory strategies when the body is destabilized. In addition, the sample size should also be enlarged in future studies.

Understanding the deficits of APAs and its consequences on postural control in the FOA population is important, and it has clinical implications in daily living activities management and in rehabilitation programs, as aged patients usually fall when moving (dynamic equilibrium) rather than during orthostatic equilibrium. As we observed, FOAs seem to have lost the programing ability to modify APA features (i.e., muscle selectivity, timing activation) under different equilibrium constraints. Reduced or non-optimal utilization of APAs in FOAs to execute daily tasks may be one contributory element to functional decline and subsequent falls. Clinical assessment for this population should include postural stability during rehabilitation programs and perhaps an APA-focused training should be included in randomized controlled trials to investigate its contribution in reducing the probability of new fall episodes in FOAs.

## 5. Conclusions

Our results are consistent with the interpretation that frail older adults present a decrease in the predictive abilities to perform tasks with different equilibrium constraints, under this pathological aging condition. Considering this, further studies may investigate how specific training could improve postural stability in FOA patients.

## Figures and Tables

**Table 1:** Sample characteristics.

	FOA (*n* = 10)
Sex	10 M
Age (years)	72.7 ± 1.42
Height (cm)	167.5 ± 0.71
Weight (kg)	68.3 ± 7.30
Physical Activity	8 sedentary 2 insufficiently active
Berg Balance Scale	43.00 ± 5.01 (39; 46)

Values are given as the mean (SD). Sedentary (no physical activity); insufficiently active (light activities lasting 10 min/5 days a week).

**Table 2:** Comparisons among kinematic parameters.

Posture	Reaction Time (ms)	Movement Duration (ms)	Velocity (m/s)	Acceleration Time/ Movement Duration
SitSup	532.87 (44.51)	747.84 (39.85)	3.47 (0.5)	0.36 (0.04)
SitUnsup	568.65 (44.34)	772.00 (65.28)	3.62 (0.4)	0.37 (0.04)
Up	571.41 (3.13)	760.99 (25.68)	3.08 (0.3)	0.36 (0.03)
ANOVA (*p*-value)	(F(2.37) = 0.11)	(F(0.65) = 0.052)	(F(2.39) = 0.11)	(F(0.49) = 0.61)

Kinematic parameters are given as mean values; values in parentheses are the SDs. Postures: SitUnsup (sit without support); Sit Sup (sit with foot support), and Up (upright position).

**Table 3:** Comparisons among the normalized integrated electromyographic activity (EMGi).

Posture	TA (%)	SOL (%)	RF (%)	ST (%)
SitSup	14.59 (10.19)	−8.14 (3.20)	11.99 (5.71)	8.84 (5.95)
SitUnsup	18.51 (27.891)	−6.04 (3.76)	10.45 (5.03)	31.53 (13.99) *
Up	13.01 (7.96)	−7.75 (3.73)	7.98 (4.19)	9.83 (4.76)
ANOVA (*p*-value)	(F(0.25) = 0.77)	(F(0.98) = 0.38)	(F(1.62) = 0.21)	(F(19.43) < 0.0000) *

Normalized integrated electromyographic activity (EMGi) of muscles. The values of the integral parameters are the means (%), and the values in the parentheses are the SDs. * *p* < 0.0000 difference between SitUnsup and the two other postures.

## Data Availability

Data available as supplementary files.

## Supplementary Materials

The following are available online at https://www.mdpi.com/article/10.3390/healthcare9080921/s1, Table S1: relate to Figure 3.

## Abbreviations

ACC/MD	Acceleration time to total movement duration
AD	Ankle displacement
APAs	Anticipatory postural adjustments
COP	Center of pressure
CNS	Central nervous system
DEL	Deltoids anterior
EMG	Electromyographic
FOAs	Frail older adults
EMGi	Integrated EMG
MD	Movement duration
MV	Movement velocity
Fz	Reaction force
RT	Reaction time
RF	Rectus femoris
RMS	Root mean square
ST	Semitendinosus
Sit Sup	Sitting with support
SitUnsup	Sitting without support
SOL	Soleus
SD	Standard deviation
TA	Tibialis anterior
Up	Upright
